# Exercise-induced hypoalgesia after acute and regular exercise: experimental and clinical manifestations and possible mechanisms in individuals with and without pain

**DOI:** 10.1097/PR9.0000000000000823

**Published:** 2020-09-23

**Authors:** Henrik Bjarke Vaegter, Matthew David Jones

**Affiliations:** a Pain Research Group, Pain Center, Odense University Hospital, Odense, Denmark; b Department of Clinical Research, Faculty of Health Sciences, University of Southern Denmark, Odense, Denmark; c School of Medical Sciences, University of New South Wales, Sydney, Australia; d Neuroscience Research Australia, Sydney, Australia

**Keywords:** Exercise, Hypoalgesia, Pain sensitivity, Mechanisms

## Abstract

Exercise and physical activity is recommended treatment for a wide range of chronic pain conditions. In addition to several well-documented effects on physical and mental health, 8 to 12 weeks of exercise therapy can induce clinically relevant reductions in pain. However, exercise can also induce hypoalgesia after as little as 1 session, which is commonly referred to as exercise-induced hypoalgesia (EIH). In this review, we give a brief introduction to the methodology used in the assessment of EIH in humans followed by an overview of the findings from previous experimental studies investigating the pain response after acute and regular exercise in pain-free individuals and in individuals with different chronic pain conditions. Finally, we discuss potential mechanisms underlying the change in pain after exercise in pain-free individuals and in individuals with different chronic pain conditions, and how this may have implications for clinical exercise prescription as well as for future studies on EIH.

## Abstract 2

This review describes methodology used in the assessment of the manifestations of exercise-induced hypoalgesia in humans and previous findings in individuals with and without pain. Possible mechanisms and future directions are discussed.

## 1. Introduction

Exercise is guideline recommended treatment for a range of chronic pain conditions.^49^ Regular exercise and physical activity in general have well-documented positive effects on a range of physical and mental health domains including cardiovascular health, stress, mood, sleep, and sexual health.^146^ In addition, clinically important reductions in pain are often observed after 8 to 12 weeks of exercise therapy^163^; however, as little as 1 session of exercise can induce hypoalgesia. This phenomenon is known as exercise-induced hypoalgesia (EIH).^92,195^ The first observation of EIH was published 40 years ago by Black et al..^14^ During the past few decades, the number of studies investigating the effect of exercise on pain has increased dramatically, likely reflecting the increasing burden of pain as well as the recognized role of exercise in the treatment of pain.

This article will begin with a brief introduction to the methodology used in the assessment of the manifestations and mechanisms of EIH in humans. The second part of the article will present an overview of the findings from previous experimental studies investigating changes in pain perception after acute and regular exercise in pain-free individuals and in individuals with different chronic pain conditions. Possible mechanisms underlying the response to exercise in pain-free individuals and in individuals with different chronic pain conditions will also be discussed. In the last part of the article, implications for exercise prescription and future EIH studies will be addressed.

### 1.1. Assessment of exercise-induced hypoalgesia—methodological considerations

The effect of a single bout of exercise on pain perception in humans has primarily been investigated experimentally in laboratory settings. The methods used in these investigations are diverse, incorporating different study designs and methods of pain assessment. Most often, EIH has been investigated using a within-group pre-post design, whereby participants' pain is assessed at different exercising and nonexercising body sites before and during/after exercise.^130^ Controlled studies using similar methodology but different designs (eg, crossover trials and parallel trials) have also been conducted.^80,162,195,204^ The results of these studies, especially those where participants were randomized to exercise or control, or where the order of exercise and control were randomized and counterbalanced for crossover trials, give a less biased estimate of the effect of a single bout of exercise on pain.

#### 1.1.1. Pain threshold, intensity, and tolerance

Pain has been quantified in a variety of ways in studies of EIH, with quantitative sensory testing used most often. Quantitative sensory testing describes a series of tests that measure the perceptual responses to systematically applied and quantifiable sensory stimuli (usually pressure, thermal, or electrical).^23^ These tests typically involve the assessment of a person's pain threshold or pain tolerance which are, respectively, the minimum intensity of a stimulus that is perceived as painful and the maximum intensity to a noxious stimulus that the participant is willing to tolerate.^116^ Ratings of pain intensity and unpleasantness during exposure to various noxious stimuli might also be measured. As an example, pressure may be applied at an increasing intensity over the lower leg using an inflated cuff, with participants asked to rate the point at which this pressure becomes painful (threshold) and then endure it for as long as possible (tolerance) while rating its intensity or unpleasantness. Using this example, EIH could manifest as an increase in pain threshold, an increase in pain tolerance, and/or a reduction in ratings of pain intensity or unpleasantness. These measures are most commonly assessed in the immediate postexercise period (eg, 0–15 minutes), but some studies have measured pain 30 to 60 minutes after exercise cessation to investigate the persistence of EIH.^69,103^

#### 1.1.2. Pain modulatory mechanisms

Methods that assess an individual's ability to modulate pain have been increasingly used in recent studies of EIH. These include temporal summation, spatial summation, conditioned pain modulation, and offset analgesia. Of these paradigms, temporal summation and conditioned pain modulation have been used most often. Temporal summation refers to an increase in pain after repetitive stimulation at the same intensity^137^ and is considered a behavioural correlate of wind-up—the frequency-dependent increase in C-fibre-evoked responses of dorsal horn neurons after repetitive stimulation at a constant intensity.^63^ Temporal summation paradigms provide information mostly about facilitatory mechanisms underlying nociceptive processes.^23^ By contrast, conditioned pain modulation provides an index of the strength of pain inhibition. Conditioned pain modulation (ie, “pain inhibits pain”) involves the application of 2 noxious stimuli over 2 different areas of the body, with the more pronounced noxious stimulus (conditioning stimulus) subsequently inhibiting the perception of the weaker noxious stimulus (test stimulus).^211,212^ Using these paradigms, EIH would manifest as a reduction in temporal summation and/or an increase in conditioned pain modulation, although evidence for the latter is limited.^2,36,122^

#### 1.1.3. Nociceptive processing

Although not an assessment of pain per se, techniques that assess the function of the nociceptive pathways have sometimes been used to investigate EIH.^38,80^ These more complex methods, which include evoked potentials and neuroimaging, may provide greater insight into the mechanisms of EIH compared to more commonly used quantitative sensory tests. Evoked potentials are cortical responses recorded at the scalp using electroencephalography in response to brief and intense stimuli. Evoked potentials are described by their polarities (negative [N] and positive [P]), latencies, and amplitudes, and consist of early, late, and ultra-late components. When analysing pain-related evoked potentials, the peak-to-peak amplitude of the N2P2 is the component most related to nociception, whereby larger N2P2 amplitude is associated with more pain.^71^ There is evidence that both the sensory-discriminative and affective aspects of pain are captured by this late component of the evoked potential, and studies have shown exercise to reduce the amplitude of this component.^72,145^ Neuroimaging is widely used in the study of pain, but to the best of our knowledge, only 2 studies have used neuroimaging to investigate acute EIH.^38,165^ In one study, brain responses to noxious thermal stimuli before and after rest and exercise were measured using functional magnetic resonance imaging in women with fibromyalgia and healthy pain-free controls. The results suggested that, in the women with fibromyalgia, exercise-stimulated brain regions involved in descending pain inhibition which, in turn, was associated with lower pain ratings to thermal stimuli.^38^ In the second study, brain responses to noxious thermal stimuli before and after walking and running exercises were measured using functional magnetic resonance imaging in 20 athletes. The results suggested that running exercise reduced the pain-induced activation in the periaqueductal gray, a key area in descending pain inhibition which, in turn, was associated with lower pain unpleasantness ratings to thermal stimuli.^165^ Taken together, these results provide evidence that a single bout of exercise can modulate pain-related areas of the nervous system.

In addition to the different study designs and techniques used to quantify pain in investigations of EIH, the exercise protocols have also varied considerably. Aerobic and isometric exercise have been studied most often,^130^ whereas dynamic resistance exercise has not commonly been used. Within each mode of exercise, the prescription has varied too. For example, aerobic exercise has consisted of cycling, running, and stepping of various durations (30 seconds–30 minutes) and intensities (low to high).^69,129,195^ The same is true of isometric exercise where upper-limb and lower-limb exercise of both short and long duration (<5 seconds—exhaustion) and varied intensity (10%–100% MVC) have been studied.^64,195^ Studies of dynamic resistance exercise have typically used whole-body training at moderate intensities.^17,93^ Interestingly, EIH is reproducible with each type of exercise, even when modest doses are used.^129,162^ This is described in more detail below.

## 2. Pain outcomes after acute and regular exercise in pain-free individuals

As illustrated in Table 1, a single session of exercise has repeatedly been observed to reduce pain sensitivity in pain-free individuals. Hypoalgesia after aerobic exercises (eg, bicycling or running), dynamic resistance exercises (eg, circuit training), and isometric exercises (eg, a wall squat) often produces an increase in pressure pain thresholds at exercising body areas of 15% to 20% compared with a quiet rest control condition.^192,200^ Increases in pain thresholds can also be observed at nonexercising body areas, although larger hypoalgesic responses are consistently observed in areas closer to the exercising muscles compared with nonexercising muscle areas. The observed EIH response is short-lasting, often with a duration lasting from 5 minutes after exercise^69^ to 30 minutes after exercise^88^ and may depend on the modality of the pain test stimulus.

**Table 1:** Summary of studies investigating acute exercise-induced hypoalgesia in pain-free individuals.

Exercise type	Exercise form	Intensity	Duration	# of partici pants	Pain test modality	Pain outcome	Local site	Remote site	Findings	Year	Author
Aerobic	Bicycling	70% HRmax	30 min	10	Chemical	Pain intensity	Thigh	—	↑Pain intensity (hyperalgesia)	1984	Vecchiet et al.^205^
Aerobic	Bicycling	50%–70% HRmax	20 min	91	Cold	CPI	—	Hand	No hypoalgesia	1992	Padawer and Levine^143^
Aerobic Aerobic	Bicycling Bicycling	70%–75% VO_2_max VO_2_max test	6 min 8–12 min	41 25	Cold Cold	CPT CPTol CPI	— —	Hand Arm	↑CPT ↑CPTol ↓CPI	2013 2018	Pokhrel et al.^154^ Chretien et al.^18^
Aerobic Aerobic	Bicycling Bicycling	50 W 100 W 150 W 200 W Increasing to 300W	Max 8 min/step 15–30 min	6 7	Electrical Electrical	EPT EPT	— —	Tooth Tooth	↑EPT ↑EPT	1984 1985	Pertovaara et al.^149^ Kemppainen et al.^87^
Aerobic	Bicycling	HR = 150/min	20 min	11	Electrical	EPT	—	Tooth	↑EPT	1986	Olausson et al.^140^
Aerobic	Bicycling	Increasing to 300 W	Unknown	6	Electrical	EPT	—	Tooth	↑EPT	1986	Kemppainen et al.^86^
Aerobic	Bicycling	Increasing to 200 W	Unknown	6	Electrical	EPT	—	Tooth	↑EPT	1990	Kemppainen et al.^88^
Aerobic	Bicycling	Increasing to 250 W	Fatigue	10	Electrical	EPT	—	Tooth Hand	↑EPT tooth ↑EPT hand	1991	Droste et al.^30^
Aerobic	Bicycling	Increasing to VO_2_max	Unknown	17	Electrical	EPT EPTol	—	Hand	↑EPT ↑EPTol	2005	Drury et al.^33^
Aerobic Aerobic Aerobic	Bicycling Bicycling Bicycling	1 KP 60 W Increasing to 200 W	5 min 10 min Unknown	60 21 28	Heat Heat Heat	HPI TSPh HPI HPI	— — —	Foot Lower leg Hand Forearm Hand Hand	↓HPI lower extremity ↓TSPh (lower extremity) ↓HPI ↓HPI	2006 2014 2019	George et al.^50^ Ellingson et al.^36^ St-Aubin et al.^175^
Aerobic	Bicycling	75% VO_2_max	30 min	16	Pressure	PPT PPI	—	Hand	↑PPT ↓PPI	1996	Koltyn et al.^95^
Aerobic Aerobic	Bicycling Bicycling	75% VO_2_max 1. 75% VO_2_max 2. 50% VO_2_max	30 min 1. 10 min 1. 20 min 2. 10 min 2. 20 min	20 80	Pressure Pressure	PPI PPT	— Thigh	Hand Arm Shoulder	No hypoalgesia ↑PPTs After 75% VO_2_max (10 and 20 min)	2006 2014	Monnier-Benoit and Groslambert^128^ Vaegter et al.^195^
Aerobic	Bicycling	75% VO_2_max	15 min	56	Pressure	PPT	Thigh	Shoulder	↑PPTs	2015	Vaegter et al.^198^
Aerobic	Bicycling	75% VO_2_max	15 min	56	Pressure	PPTol TSPp	Lower leg	Arm	↑PPTol lower leg ↓TSPp lower leg	2015	Vaegter et al.^196^
Aerobic	Bicycling	1. 75% VO_2_max 2. 50% VO_2_max	20 min	80	Pressure	PPTol TSPp	Lower leg	Arm	No hypoalgesia	2015	Vaegter et al.^196^
Aerobic	Bicycling	1. 70% VO_2_max 2. 30% VO_2_max	30 min	10	Pressure	PPT	Thigh	Forearm	↑PPT thigh After 70% VO_2_max ↓PPT thigh and arm After 30% VO_2_max (hyperalgesia)	2016	Micalos and Arendt-Nielsen^124^
Aerobic	Bicycling	Increasing to VO_2_max	Fatigue	50	Pressure	PPT	Knee	Ankle Arm Chest Head	↓PPT Chest (hyperalgesia) ↓PPT Head (hyperalgesia)	2016	Kruger et al.^103^
Aerobic	Bicycling	RPE = 14–15	20 min	40	Pressure	PPT	Thigh	Shin Hand	↑PPT thigh ↑PPT shin ↑PPT hand	2017	Jones et al.^81^
Aerobic	Bicycling	RPE = 17	5 min	36	Pressure	PPT	Thigh	Hand	↑PPT thigh ↑PPT hand	2017	Jones et al.^79^
Aerobic	Bicycling	RPE = 16	15 min	34	Pressure	PPT	Thigh	Shoulder	↑PPT thigh ↑PPT shoulder	2018	Vaegter et al.^193^
Aerobic	Bicycling	1. HIIT: 90%–100% of max workload 2. MICT: 65%–75% of HR	1. 10 × 1 min 2. 30 min	28	Pressure	PPT	Thigh	Shin Shoulder	No hypoalgesia	2018	Hakansson et al.^58^
Aerobic	Bicycling	75% VO_2_max	15 min	31	Pressure	PPT	Thigh	Back Hand	↑PPT thigh ↑PPT back ↑PPT hand	2018	Gajsar et al.^45^
Aerobic Aerobic	Bicycling Bicycling	50 W 75% VO_2_max	12 min 15 min	20 30	Pressure Pressure	TSPp PPT	Thigh Thigh	Shoulder Back Hand	↓TSPp trapezius ↑PPT thigh ↑PPT back	2018 2019	Malfliet et al.^118^ Gomolka et al.^54^
Aerobic	Bicycling	Lactate threshold	15 min	34	Pressure	PPT	Thigh	Shoulder	↑PPT thigh	2019	Vaegter et al.^192^
Aerobic	Bicycling	75%–88% HRmax	20 min	15	Pressure Electrical	PPT EPI	Thigh	Shoulder Thoracic spine Hand Esophagus	No hypoalgesia	2017	van Weerdenburg et al.^204^
Aerobic	Bicycling	1. 70% HR max 2. 86% HR max	1. 24 min 2. 4 × 4 min	29	Pressure Heat	PPT HPT HPI	—	Hand	↓HPI after interval condition	2014	Kodesh and Weissman-Fogel^91^
Aerobic	Bicycling	1. 70% HRR 2. 50%–55% HRR	20 min	27	Pressure Heat	PPT PPI HPI TSPh	—	Forearm	↑PPT after high intensity ↓HPI ↓TSPh	2014	Naugle et al.^132^
Aerobic	Bicycling	Intensity = pain level 3/10	15 min	16	Pressure Heat	PPT HPT	Thigh	Hand	↑PPT ↑HPT	2016	Black et al.^11^
Aerobic	Bicycling	1. 75% VO_2_max 2. 50% VO_2_max	25 min	43	Pressure Heat	PPT PPI HPI TSPh	Forearm	Forearm	↑PPTs	2016	Naugle et al.^133^
Aerobic	Bicycling	60–70 W	20 min	40	Pressure Heat	PPT HPT TSPh	Achilles	—	No hypoalgesia	2016	Stackhouse^176^
Aerobic	Bicycling	70% HRR	15 min	16	Pressure Heat	PPT HPT HPI	Thigh	Shin Foot	↑PPT thigh ↑PPT shin ↓HPI foot	2019	Jones et al.^78^
Aerobic	Bicycling	200 W	20 min	6	Reflex	NFR	Thigh	—	↑NFR	1992	Guieu et al.^55^
Aerobic	Repeated back movements	Lifting 5 kg	7 min	18	Pressure Heat Cold	PPT HPT CPT TSPp	Back	Hand	↑PPT back ↑CPT hand	2019	Kuithan et al.^104^
Aerobic	Running	Near anaerobic threshold	30 min	27	Cold	CPT CPI	—	Hand	↑CPT	2011	Wonders and Drury^210^
Aerobic	Running	Unknown	30 min	22	Heat	HPI	—	Forearm	No hypoalgesia	1993	Fuller and Robinson^44^
Aerobic	Running	Self-selected	40 min	1	Pressure	PPT PTT	—	Arm	↑PPT ↑PTT	1979	Black et al.^14^
Aerobic	Running	Self-selected	1 mile	15	Pressure	PPT	—	Hand	↑PPT hand	1981	Haier et al.^57^
Aerobic	Running	VO_2_max test	Unknown	29	Pressure	PPI	—	Arm	↓PPI	2001	Oktedalen et al.^139^
Aerobic	Running	1. 75% VO_2_max 2. 75% VO_2_max 3. 50% VO_2_max	1. 10 min 2. 30 min 3. 10 min	12	Pressure	PPI	—	Hand	↓PPI after 30 min at 75% VO_2_max	2004	Hoffman et al.^69^
Aerobic	Running	65%–75% of HRR	7 min	12	Pressure	PPT	—	Forearm	↑PPT	2004	Drury et al.^32^
Aerobic	Running	Unknown	100 mile	30	Pressure	PPI	—	Hand	↓PPI	2007	Hoffman et al.^67^
Aerobic	Running	VO_2_max test	Unknown	62	Pressure	PPT	Thigh	Shoulder Hand	↑PPT	2015	Stolzman et al.^179^
Aerobic	Running	110% Gas exchange threshold	30 min	26	Pressure	PPT	Thigh	Forearm	↑PPT forearm ↑PPT thigh	2019	Peterson et al.^151^
Aerobic	Running	85% VO_2_max	44 min	12	Pressure Heat Cold	PPI HPI CPI CPT	—	Hand Arm	↓HPI ↓PPI	1984	Janal et al.^74^
Aerobic	Running	85% HRmax	10 min	63	Heat Cold	HPT CPI	—	Hand Forearm	↓HPT (hyperalgesia) ↓CPI	2001	Sternberg et al.^178^
Aerobic	Running	75% VO_2_max	30 min	14	Heat Cold	HPT CPT HPI CPI	—	Hand	No hypoalgesia	2005	Ruble et al.^159^
Aerobic	Step	63% VO_2_max	12 min	60	Pressure	PPI PTT	—	Hand	↓PPI ↑PTT	1994	Gurevich et al.^56^
Aerobic	Step	50% of maximum number of steps in 1 minute	5 min	30	Pressure	PPI TSPp	—	Forearm	↓PPI ↓TSPp	2019	Nasri-Heir et al.^129^
Aerobic Aerobic	Walking Walking	6.5 km/h Fast walking	10 min 40 min 6 min	5 35	Pressure Pressure	PPT PPTol	Thigh Calf	Shoulder Shoulder	No hypoalgesia ↑cPTT Calf	2014 2019	Lee^110^ Hviid et al.^73^
Anaerobic	Wingate test	“All-out”	30 seconds	50	Pressure	PPT	—	Shoulder Jaw	↓PPTs (hyperalgesia)	2012	Arroyo-Morales et al.^3^
Anaerobic Anaerobic	Bicycle Sprint Wingate test	“All-out” “All-out”	3 × 6 seconds 30 seconds	12 50	Pressure Pressure Heat	PPT PPT HPT TSPh TSPc	Thigh Thigh	Lower leg Hand	↓PPTs (hyperalgesia) ↑PPT thigh ↑HPT hand ↓TSPh hand ↓TSPc hand	2018 2018	Klich et al.^89^ Samuelly-Leichtag et al.^162^
Dynamic resistance	Full-body circuit	Moderate	20 min	17	Pressure	PPT PPTol	Shin	—	↑PPTol	1996	Bartholomew et al.^5^
Dynamic resistance	Full-body circuit	75% 1RM	4 exercises 3 × 10 repetitions (45 min)	13	Pressure	PPT PPI	—	Hand	↑PPT ↓PPI	1998	Koltyn and Arbogast^93^
Dynamic resistance	Full-body circuit	75% 1RM	4 exercises 3 × 10 repetitions (45 min)	21	Pressure	PPT PPI	—	Hand	↑PPT ↓PPI	2009	Focht and Koltyn^42^
Dynamic resistance Dynamic resistance	Upper-body circuit Full-body circuit	Unknown 60% 1RM	10 min 40 min 3 exercises 12 repetitions	5 24	Pressure Pressure	PPT PPT PPTol	Shoulder —	— Hand	No hyperalgesia ↑PPTol	2014 2017	Lee^110^ Baiamonte et al.^4^
Dynamic resistance Dynamic resistance	Kettlebell swings Full-body circuit	8–12 kg 60% 1RM	8 × 20 seconds 9 exercises 12 repetitions	32 10	Pressure Pressure	PPT PTT	Lower back Buttock Hand	— —	↑PPTs ↑PTT hand	2017 2018	Keilman et al.^84^ McKean et al.^119^
Dynamic resistance	Handgrip	100% MVC	30 contractions in 1 minute	12	Pressure	PPT	—	Forearm	↑PPT	2004	Drury et al.^32^
Dynamic resistance	Handgrip	Medium	Maximum of 40 contractions in 1 minute	48	Heat	HPI	—	Hand	↓HPI	2008	Weissman-Fogel et al.^207^
Dynamic ``Resistance	Back extensions	Bodyweight	3 × 15 repetitions	20	Heat	HPI TSPh	—	Foot Lower leg Hand Forearm	↓HPI (lower extremity)	2006	George et al.^50^
Dynamic resistance	Cervical flexions	Head weight	3 × 10 repetitions	30	Pressure Heat	PPT HPI TSPh	—	Foot Hand	↑PPT ↓HPI	2011	Bishop et al.^10^
Eccentric Eccentric Eccentric	Wrist extension Elbow flexion Heel-raise	30% MVC Max Bodyweight	5 × 10 repetitions 10 × 6 repetitions 4 × 15 contractions	13 10 40	Pressure Pressure Electrical Pressure Heat	PPT PPT EPT PPT HPT TSPh	Forearm Arm Achilles	— — —	↑PPT ↓PPT ↓EPT (hyperalgesia) PPT ↓TSPh	2010 2015 2016	Slater et al.^171^ Lau et al.^108^ Stackhouse et al.^176^
Isometric	1. Knee extension 2. Elbow flexion	1. 30% MVC 2. 60% MVC	1. 90 seconds 1. 180 seconds 2. 90 seconds 2. 180 seconds	80	Pressure	PPT	Thigh (knee extension) Arm (elbow flexion)	Shoulder	↑PPTs After low and high intensity exercises	2014	Vaegter et al.^195^
Isometric	1. Knee extension 2. Elbow flexion	1. 30% MVC 2. 60% MVC	3 min	80	Pressure	PPTol TSPp	Lower leg	Arm	↑PPTol (after both elbow and knee exercises) ↓TSPp arm and leg (after low and high intensity exercises)	2015	Vaegter et al.^196^
Isometric	1. Knee extension 2. Elbow flexion	20% of MVC	Fatigue	64	Pressure	PPT PPI	—	Hand	↑PPT after elbow flexion (women only)	2016	Lemley et al.^113^
Isometric	1. Knee extension 2. Shoulder rotation	1. 1 kg 2. 0.5 kg	Fatigue	24	Pressure	PPT	Thigh Shoulder	Shoulder `Thigh	↑PPT thigh + shoulder both conditions	2003	Kosek and Lundberg^101^
Isometric	Back extension	—	2 min	29	Pressure	PPT	Back	Thigh Hand	↑PPT thigh ↑PPT hand (women)	2017	Gajsar et al.^46^
Isometric	Elbow flexion	1. Max contractions 2. 25% MVC 3. 25% MVC 4. 80% MVC	1. 3 reps 2. Fatigue 3. 2 min 4. Fatigue	40	Pressure	PPT PPI	—	Hand	↑PPT and ↓PPI after max and after 25% MVC until fatigue	2008	Hoeger Bement et al.^64^
Isometric	Elbow flexion	25% MVC	Fatigue	20	Pressure	PPT PPI	Hand	—	↑PPT ↓PPI	2009	Hoeger Bement et al.^65^
Isometric	Elbow flexion	25% MVC	Fatigue	26	Pressure	PPT PPI	Hand	—	↑PPT ↓PPI (men only)	2014	Bement et al.^7^
Isometric	Elbow flexion	1. Max contractions 2. 25% MVC 3. 25% MVC	1. 3 reps 2. Fatigue 3. 2 min	24	Pressure	PPT PPI	Hand	—	↑PPT ↓PPI (women only)	2014	Lemley et al.^111^
Isometric	Elbow flexion	25% MVC	Fatigue	39	Pressure	PPI	Hand	—	↓PPI	2014	Lemley et al.^112^
Isometric	Elbow flexion	40% MVC	3 min	26	Pressure Heat	PPT HPT	Arm	Hand	↑PPTs	2016	Jones et al.^80^
Isometric	Arm abduction	1 kg	Fatigue	25	Pressure	PPT	Shoulder	Shoulder	↑PPTs	2000	Persson et al.^147^
Isometric	Handgrip	25% MVC	2 min	134	Cold	CPT CPI	—	Hand	↑CPT hand	2017	Foxen-Craft and Dahlquist^43^
Isometric	Handgrip	25% MVC	3 min	34	Electrical	EPI	—	Lower leg	↓EPI	2016	Umeda et al.^188^
Isometric	Handgrip	1. 40% MVC 2. 25% MVC	1. Fatigue 2. 3 min	88	Heat	TSPh	Hand	—	↓TSPh for both conditions	2013	Koltyn et al.^96^
Isometric	Handgrip	1. Maximal 2. 40%–50% MVC	2 min	31	Pressure	PPT PPI	Hand	—	↑PPT ↓PPI	2001	Koltyn et al.^97^
Isometric	Handgrip	40%–50% MVC	2 min	40	Pressure	PPT PPI	Hand	Hand	↑PPT both sites ↓PPI both sites	2007	Koltyn and Umeda^98^
Isometric Isometric	Handgrip Handgrip	33% MVC 1. 25% MVC 2. 25% MVC	3 min 1. 1 minute 2. 3 min	79 23	Pressure Pressure	PPTol PPT PPI	Hand Hand	— —	↑PPTol No hypoalgesia	2009 2009	Alghamdi and Al-Sheikh^1^ Umeda et al.^190^
Isometric	Handgrip	25% MVC	1. 1 minute 2. 3 min 3. 5 min	50	Pressure	PPT PPI	Hand	—	↑PPT and ↓PPI after all durations	2010	Umeda et al.^189^
Isometric	Handgrip	50% MVC	Fatigue	50	Pressure	PPT	Forearm	Forearm	↑PPT	2017	Black et al.^12^
Isometric	Handgrip	50% MVC	Fatigue	26	Pressure	PPT	Forearm	Thigh	↑PPT forearm ↑PPT thigh	2019	Peterson et al.^151^
Isometric	Handgrip	1. 1% MVC 2. 15% MVC 3. 25% MVC	Unknown	2008	Electrical Reflex	EPI NFR	—	Lower leg	↓EPI after 15% and 25% MVC	2008	Ring et al.^157^
Isometric	Handgrip	25% MVC	3 min	27	Pressure Heat	PPT PPI HPI TSPh	Forearm	Forearm	↑PPT ↓HPI (women) ↓TSPh	2014	Naugle et al.^131^
Isometric	Handgrip	25% MVC	3 min	58	Pressure Heat	PPT PPI TSPh	Hand	—	↑PPT ↓PPI ↓TSPh	2014	Koltyn et al.^94^
Isometric	Handgrip	25% MVC	3 min	43	Pressure Heat	PPT PPI HPI TSPh	Forearm	Forearm	↑PPT ↓TSPh	2016	Naugle et al.^133^
Isometric	Handgrip	25% MVC	3 min	58	Pressure Heat	PPT PPI TSPh	Hand	—	↑PPT ↓PPI ↓TSPh	2017	Brellenthin et al.^16^
Isometric	Handgrip	25% MVC	3 min	58	Pressure Heat	PPI HPI	Hand	—	↓PPI hand ↓HPI hand	2018	Crombie et al.^22^
Isometric	Handgrip	25% MVC	3 min	52	Pressure Heat	PPT HPI	—	Forearm	↓PPT (hyperalgesia)	2018	Ohlman et al.^138^
Isometric	Knee extension	21% MVC	Fatigue	14	Pressure	PPT	Thigh	—	↑PPT	1995	Kosek and Ekholm^99^
Isometric	Knee extension	30% MVC	Fatigue	134	Pressure	PPT	—	Shoulder	↑PPT	2017	Tour et al.^185^
Isometric	Knee extension	0.75 kg	12 min	15	Pressure Electrical	PPT EPI	Thigh	Shoulder Thoracic spine Hand Esophagus	No hypoalgesia	2017	van Weerdenburg et al.^204^
Isometric	Knee extension	30% MVC	3 min	20	Pressure Heat	PPT PPTol HPT	—	Lower leg	↑PPTol	2017	Vaegter et al.^199^
Isometric	Knee extension	20%–25% MVC	5 min		Pressure Heat	PPT PPI HPI	Shin	Neck	↑PPT shin	2018	Harris et al.^61^
Isometric	Pinch grip	25% MVC	15 seconds	38	Heat	HPI	Hand	Hand	No hypoalgesia	2013	Paris et al.^144^
Isometric	Pinch grip	1. 5% MVC 2. 25% MVC 3. 50% MVC	15 seconds	42	Heat	HPI	Hand	Hand	↓HPI with larger effects for higher intensity	2014	Misra et al.^127^
Isometric	Teeth-clenching	—	Fatigue	33	Pressure	PPT	Jaw	Forearm	↑PPT jaw	2019	Lanefelt et al.^106^
Isometric	Trunk flexion	—	Fatigue	70	Pressure	PPT	Abdomen	Nailbed	↑PPT Abdomen	2019	Deering et al.^27^
Isometric	Wall squat	—	3 min	35	Pressure	PPT	Thigh	Shoulder	↑PPT thigh ↑PPT shoulder	2019	Vaegter et al.^200^

The table is organized according to exercise type, exercise form, pain test modality, and year of publication.

CPI, cold pain intensity; CPT, cold pain threshold; EPI, electrical pain intensity; EPT, electrical pain threshold; EPTol, electrical pain tolerance; HIIT, high-intensity interval training; HPI, heat pain intensity; HPT, heat pain threshold; HRmax, maximum heart rate; HRR, heart rate reserve; MICT, moderate-intensity continuous training; MVC, maximal voluntary contraction; NFR, nociceptive flexion reflex; PPI, pressure pain intensity; PPT, pressure pain threshold; PPTol, pressure pain tolerance; RM, repetition maximum; RPE, rating of perceived exertion; TSPc, temporal summation of cold pain; TSPh, temporal summation of heat pain; TSPp, temporal summation of pressure pain; VO_2_max, maximal aerobic capacity.

### 2.1. Exercise intensity and duration

The hypoalgesic responses seem to be similar between exercise types,^133,195^ although EIH differences have been observed,^32^ but exercise intensity and duration quite consistently affect the EIH response. Exercise intensity affects the EIH response after aerobic exercise.^69,124,132,195^ For example, in 80 pain-free individuals, it was observed that a moderate-to-high intensity bicycling exercise produced significantly larger EIH responses at the exercising quadriceps muscle, as well as at the nonexercising biceps and trapezius muscles, compared with a low-intensity bicycling exercise.^195^ Findings on the influence of aerobic exercise duration are more equivocal, with one study observing a dose-response with larger effects after bicycling for 30 minutes compared with 10 minutes,^69^ and one study observing no difference between bicycling for 10 minutes compared with 20 minutes.^195^ Moreover, the fact that very short-duration aerobic exercise can elicit EIH^129,162^ implies that intensity, or the combination of intensity and duration, may be more important for determining the size of EIH after aerobic exercise than either variable alone.

Exercise intensity and duration may also affect the EIH response after isometric exercises,^64,127,157^ although the results are more inconsistent. In 40 individuals, pressure pain thresholds at the hand were increased and pressure pain intensity was decreased after low-intensity (25% of maximal voluntary contraction [MVC]) isometric elbow flexion until exhaustion. However, no hypoalgesia was observed when the contraction was held for only 2 minutes.^64^ By contrast, hypoalgesia was found after 90 and 180 seconds isometric knee extensions and elbow flexion exercises at 30% MVC and 60%, respectively, in 80 healthy individuals; however, the hypoalgesic responses were not different in magnitude between low-intensity and high-intensity contractions nor between shorter or longer durations.^195^ The fact that very low doses of isometric exercise (eg, three maximal contractions of 5-second duration, totaling 15 seconds of exercise) can produce EIH^64^ lends further support to the lack of clear dose-response, which is further evidenced by a study of 50 individuals where elevations in pain threshold were not different between isometric handgrip exercises at 25% MVC for 1, 3, or 5 minutes.^189^

### 2.2. Effects on pain modulatory mechanisms

As described, robust increases in pressure pain thresholds are observed after exercise, but exercise can also affect spinal and supraspinal mechanisms of pain. Temporal summation of pressure and heat pain was reduced after submaximal isometric exercises at 25% to 40% of MVC for 3 minutes,^94,96,131,196^ and 20 minutes of aerobic exercise at 55% to 70% of heart rate reserve reduced temporal summation of heat pain^132^; however, temporal summation of pressure pain was not affected by 15 to 20 minutes of aerobic exercise at 50% to 75% of VO2max.^196^ However, not all studies have shown exercise to have positive effects on pain mechanisms. For example, Alsouhibani et al. observed a decrease in the CPM response after exercise.^2^ By contrast, other studies have found exercise to have no effect on CPM^122^ or offset analgesia,^61^ suggesting that exercise can, but does not always, influence spinal and supraspinal mechanisms of pain. Exercise can also influence the ability to cope with pain. The perceived pain intensity of a suprathreshold stimulus is consistently reduced by aerobic, isometric, and dynamic resistance exercises,^42,64,98^ and acute exercise can reduce ratings of pain unpleasantness even in the absence of a change in pain intensity.^80^ In addition, low-intensity nonpainful aerobic and isometric exercises also increase the tolerance to a painful stimulus. A 20% increase in pain tolerance was observed by Vaegter et al.^199^ after a 3-minute submaximal isometric knee extension exercise, and after a 6-minute walking exercise^73^ compared with rest in 35 pain-free individuals.

### 2.3. Factors influencing exercise-induced hypoalgesia

Exercise that produces acute hypoalgesia is often perceived as moderately painful with peak pain intensity ratings around 5 or 6 on a 0 to 10 numerical rating scale,^193,200^ and painful exercises seem to have larger hypoalgesic effects than nonpainful exercises, at least in pain-free individuals,^36^ but perhaps not in individuals with chronic pain.^20,173^

Treatment expectations are a well-recognized factor known to modulate treatment outcomes and the information about the effect of exercise given to individuals before exercise influences the magnitude of the EIH response. First, a randomized controlled trial by Jones et al.^81^ observed that the hypoalgesic effect after bicycling was slightly increased if positive information about EIH was given before the exercise compared to when no EIH information was given before exercise. Second, a randomized controlled trial by Vaegter et al. comparing positive vs negative pre-exercise information observed a 22% increase in pain thresholds in the positive information group, whereas the negative information group had a 4% decrease (hyperalgesia) in pain threshold at the exercising muscle (Vaegter et al., in review). Both studies observed a positive correlation between expectations and hypoalgesia after exercise.

Despite robust hypoalgesia after exercise on a group level, the response to exercise is not identical across individuals and across days. Several studies have investigated the stability of the EIH response in pain-free individuals across different days using a number of aerobic^54,73,192,193^ and isometric^200^ exercise protocols. Across protocols, some individuals consistently show hypoalgesia after exercise, some individuals consistently showed hyperalgesia after exercise, and some individuals had a change in their response from hypoalgesic to hyperalgesic or vice versa between days. Interestingly, the majority of individuals showed hypoalgesia at some point.

### 2.4. Regular exercise and pain

The effect of regular exercise and physical activity on pain sensitivity has been investigated, albeit less than the effect of a single session of exercise. In pain-free individuals, there have been relatively few studies investigating whether those who are more physically active experience greater EIH. The results of these studies show that EIH is usually similar between inactive and active pain-free individuals irrespective of the type of exercise they regularly perform (ie, aerobic or strength training) and the methods used to assess physical activity (ie, self-report or objectively measured using accelerometry).^12,188,198^ However, Ellingson et al.^35^ observed lower pain intensity ratings and lower pain unpleasantness ratings to suprathreshold heat pain stimulations in pain-free women who were physically active as defined by the current public health recommendations compared with women who were less physically active than recommended. There is also some evidence that individuals who are more physically fit experience greater EIH.^138,166^

Regarding the effect of a longer period of exercise training in pain-free individuals, Hakansson et al.^58^ observed changes in PPT in the legs after 6 weeks of moderate bicycling exercises (3 times/week) but not after high-intensity interval exercise. In addition, Jones et al.^76^ observed increases in pressure pain tolerance but not pain threshold after bicycling 30 minutes at 75% of VO_2_ max 3 times/week for 6 weeks compared with a control condition. These findings suggest that regular exercise in pain-free individuals specifically influences the ability to cope with pain (ie, pain perception above the pain threshold) rather than the level at which pain is first perceived (pain threshold). Similar observations have been found in athletes compared with less active individuals. A systematic review with meta-analysis by Tesarz et al.^182^ showed consistently higher pain tolerance across different pain modalities (ie, pressure, heat, cold, electrical, and ischemic) in athletes; however, for pain thresholds, the conclusion was less consistent.

In addition to the effect on pain tolerance, regular exercise may also affect the ability to inhibit pain as assessed by the CPM paradigm. Naugle et al. observed that pain-free individuals reporting more regular physical activity also had a larger CPM response compared with individuals reporting less regular physical activity.^134,135^ Although previous investigations on CPM in athletes have been equivocal because increased CPM^52^ as well as decreased CPM^181^ has been observed, the positive effect of regular exercise on CPM may be a potential mechanism underlying the preventive effect of exercise on pain because better CPM capacity has been associated with a reduced risk of chronic pain.^211^ The preventive effect of regular exercise is supported by a recent systematic review with meta-analysis concluding that regular exercise performed 2 to 3 times/week reduces the risk of low back pain by 33%.^169^ This is true even in those who are at an increased risk of developing chronic pain.^115^

## 3. Pain outcomes after acute and regular exercise in individuals with chronic pain

In individuals with different chronic pain conditions, the response to a single session of exercise is less consistent as hypoalgesia, reduced hypoalgesia, or even hyperalgesia (ie, increased sensitivity to pain) has been observed. As illustrated in Table 2, hypoalgesia after exercise has, eg, been observed in individuals with chronic musculoskeletal pain,^123,197^ shoulder pain,^105^ patella femoral pain,^180^ knee osteoarthritis,^59,194^ menstrual pain,^186^ and rheumatoid arthritis.^117^ However, reduced EIH responses or even hyperalgesia after exercise has often been demonstrated in individuals with whiplash-associated disorder,^203^ ME/CFS,^123,202^ fibromyalgia pain,^100,107,177^ painful diabetic neuropathy,^90^ chronic musculoskeletal pain,^19^ and also in a delayed-onset muscular soreness pain model.^25^ Hyperalgesia after exercise is often observed in individuals with more widespread chronic pain conditions. This was first observed by Kosek et al.^100^ in 5 individuals with fibromyalgia who showed a decrease in pain thresholds during and after an isometric knee extension exercise. The observation of hypoalgesia after exercise in some groups with chronic pain conditions and the observation of hyperalgesia after exercise in other groups with chronic pain may be influenced by whether the exercise is performed using a painful or nonpainful body area. Lannersten and Kosek^107^ observed hypoalgesia after a 5-minute submaximal (25% of MVC) isometric exercise in individuals with shoulder myalgia when the exercise was performed by a nonpainful leg muscle but when the exercise was performed by the painful shoulder muscle, no hypoalgesic response was observed. Similarly, Burrows et al.^17^ observed increases in pressure pain threshold after upper-body but not lower-body resistance exercise in people with knee osteoarthritis. These findings suggest that hypoalgesia can be induced by exercising nonpainful muscles in subjects with chronic pain,^191^ which may have important implications for exercise prescription in the clinical setting.

**Table 2:** Summary of studies investigating acute exercise-induced hypoalgesia in individuals with different pain conditions.

Exercise type	Exercise form	Intensity	Duration	# of participants	Pain condition	Pain test modality	Pain outcome	Local site	Remote site	Findings	Year	Author
Aerobic	Bicycling	Increasing to 75% HRmax	Unknown	20	ME/CFS	Clinical	Pain intensity	—	—	No hypoalgesia	2017	Oosterwijck et al.^141^
Aerobic	Bicycling	VO_2_max test	8–12 min	25	Chronic pain	Cold	CPI	—	Arm	No hypoalgesia	2018	Chretien et al.^18^
Aerobic	Bicycling	1 KPa	5 min	12	Chronic Low back pain	Heat	HPI TSPh	—	Forearm Lower leg	↓ TSPh forearm	2009	Bialosky et al.^9^
Aerobic	Bicycling	80% VO_2_max	30 min	23	DOMS MODEL	Pressure	PPT	—	Arm	No hypoalgesia	2002	Dannecker et al.^25^
Aerobic	Bicycling	70% VO_2_max	20 min	8	Chronic low back pain	Pressure	PPI	—	Hand	↓PPI	2005	Hoffman et al.^68^
Aerobic	Bicycling	Increasing to 130 W	37 min	26	Chronic fatigue syndrome	Pressure	PPT	Lower leg	Hand Lower back Shoulder	↓PPTs (hyperalgesia)	2010	Meeus et al.^123^
Aerobic	Bicycling	Increasing to 130 W	37 min	21	Chronic low back pain	Pressure	PPT	Lower leg	Hand Lower back Shoulder	↑PPTs	2010	Meeus et al.^123^
Aerobic	Bicycling	1. 75% HRmax 2. Self-paced	Unknown	22	hronic fatigue syndrome	Pressure	PPT	Lower leg	Hand Lower back	↑PPT lower back (after self-paced) ↓PPTs calf/hand (after self-paced) (hyperalgesia) ↓PPTs (after 75% HRmax) (hyperalgesia)	2010	Van Oosterwijck et al.^202^
Aerobic	Bicycling	1. Increasing to 75% HRmax 2. Self-paced	1. Unknown 2. Individual	20	ME/CFS	Pressure	PPT	Lower leg	Hand Lower back	No hypoalgesia/some hyperalgesia	2010	Van Oosterwijck et al.^202^
Aerobic	Bicycling	1. 62% HRmax 2. Self-paced	20 min	21	Fibromyalgia	Pressure	PPT PPI PPTol	—	Hand	↑PPT and PPTol (both conditions) ↓PPI (both conditions)	2011	Newcomb et al.^136^
Aerobic	Bicycling	1. 75% HRmax 2. Self-paced	Unknown	22	WAD	Pressure	PPT	Lower leg	Hand Lower back	↑PPT lower back (after self-paced) ↓PPTs calf/hand (after self-paced) (hyperalgesia) ↓PPTs (after 75% HRmax) (hyperalgesia)	2012	Van Oosterwijck et al.^203^
Aerobic	Bicycling	Increasing to 75% HRmax	Maximum of 15 min	19	Fibromyalgia with chronic fatigue	Pressure	TSPp	—	Shoulder Hand	No hypoalgesia	2015	Meeus et al.^122^
Aerobic	Bicycling	Increasing to 75% HRmax	Maximum of 15 min	16	RA	Pressure	TSPp	—	Shoulder Hand	No hypoalgesia	2015	Meeus et al.^122^
Aerobic	Bicycling	75% of VO_2_max	15 min	61	Chronic MSK pain	Pressure	PPT PTTol TSPp	Thigh	Arm Shoulder Lower leg	↑PPTs ↑PPTol ↑TSPp (in high pain sensitive patients)	2016	Vaegter et al.^197^
Aerobic Aerobic	Bicycling Bicycling	1. 70% HRmax 2. 75%–85% HRmax 75% of VO_2_max	1. Continuous 20 min 2. Interval 5 × 4 min 15 min	15 14	Chronic fatigue syndrome Knee OA	Pressure Pressure	PPT PTTol	Thigh Thigh	Shoulder Hand Arm Shoulder Lower leg	↑PPT thigh after interval ↑PPTs	2016 2017	Sandler et al.^164^ Vaegter et al.^194^
Aerobic	Bicycling	75% of HRmax	30 min	21	WAD	Pressure	PPT	—	Neck Shin	No hypoalgesia	2017	Smith et al.^172^
Aerobic Aerobic	Bicycling Bicycling	Increasing to 75% HRmax 50 W	Unknown 12 min	40 20	Knee OA Chronic fatigue syndrome	Pressure Pressure	PPT TSPp	Thigh Knee Thigh	Forearm Shoulder	↑PPTs (if normal CPM) ↓PPTs (if abnormal CPM) No hyperalgesia	2017 2018	Fingleton et al.^40^ Malfliet et al.^118^
Aerobic	Bicycling	70% VO_2_max	30 min	27	Gulf veterans	Pressure Heat	PPT HPI	—	Hand	↑HPI (if pain) (hyperalgesia)	2010	Cook et al.^19^
Aerobic	Running	Bruce test	Fatigue	10	Fibromyalgia	Heat	TSPh	—	Hands	↑TSPh (hyperalgesia)	2001	Vierck et al.^206^
Aerobic	Running	5 km/hour	3 × 5min	5	Chronic fatigue syndrome	Pressure	PPT	—	Hands	↓PPTs (hyperalgesia)	2004	Whiteside et al.^208^
Aerobic Aerobic	Walking Walking	Self-selected 1. Continuous 1.3 m/second 2. Interval 1.3 m/second	4 min 1. 45 min 2. 3 × 15 min	20 27	Plantar fasciopathy Knee OA	Clinical pain PPT Clinical pain	Pain intensity during test PPT Pain intensity	Heel —	— —	No hypoalgesia ↑Pain intensity continuous walking (hyperalgesia)	2018 2017	Riel et al.^156^ Farrokhi et al.^39^
Aerobic	Stepping	50% of maximum number of steps in 1 minute	5 min	30	TMD	Pressure	PPI TSPp	—	Forearm	↓TSPp	2019	Nasri-Heir et al.^129^
Dynamic resistance	Leg exercises	1. 60% 1RM 2. Self-selected	2 exercises 6 × 10 repetitions	32	Fibromyalgia	Clinical	Pain intensity	—	—	Hyperalgesia	2018	da Cunha Ribeiro et al.^24^
Dynamic resistance Dynamic resistance	Knee extensions Knee extensions	1RM 8RM	6 × 10 repetitions 1 exercise 3 × 8 repetitions	20 21	Knee OA Patellar tendinopathy	Clinical Clinical pressure	Pain intensity DOMS Pain intensity during SLS PPT	Knee Knee shin	— Forearm	No change in pain intensity More DOMS than controls ↓Pain intensity ↑PPT shin	2013 2019	Germanou et al.^51^ Holden et al.^70^
Dynamic resistance	Arm-raises	Fast	6 min	24	Knee OA	Pressure	PPT	Shoulder	Thigh	↑PPT shoulder	2020	Hansen et al.^59^
Dynamic resistance	1. Hip abductions 2. Knee extensions	Load = 12RM	3 exercises 12 repetitions	30	PFP	Pressure	PPT PTTol TSPp	Knee Lower leg	Elbow (PPT)	↑PPT (lower leg) ↑PPTol (after knee exercises)	2019	Straszek et al.^180^
Dynamic resistance	Lower-body circuit	60% 1RM	3 exercises 10 repetitions	11	Knee OA	Pressure	PPT PPTol	Thigh Knee Shin	Shoulder Arm Forearm Hand	No hypoalgesia	2014	Burrows et al.^17^
Dynamic Resistance	Upper-body circuit	60% 1RM	3 exercises 10 repetitions	11	Knee OA	Pressure	PPT PPTol	Shoulder Arm Forearm Hand	Thigh Knee Shin	↑PPTs (across sites)	2014	Burrows et al.^17^
Dynamic resistance Dynamic resistance	Back extensions Repeated back movements	Bodyweight Lifting 5 kg	3 × 15 repetitions 7 min	12 18	Chronic low back pain Chronic low back pain	Heat Pressure Heat Cold	HPI TSPh PPT HPT CPT TSPp	— Back	Forearm Lower Leg Hand	↓TSPb forearm ↑CPT hand	2009 2019	Bialosky et al.^9^ Kuithan et al.^104^
Dynamic resistance	Cervical flexion	Head weight	10 × 10 seconds	13	Chronic neck pain	Clinical pain Pressure	Pain intensity PPT	Neck	Shoulder	↓Pain intensity ↑PPTs	2018	Galindez-Ibarbengoetxea et al.^47^
Isometric	Elbow flexion	1. 25% MVC 2. 25% MVC 3. 100% MVC	1. 2 min 2. Fatigue 3. 3 reps	15	Fibromyalgia	Pressure	PPT PPI	—	Hand	No hypoalgesia	2011	Hoeger Bement et al.^66^
Isometric	Handgrip	25% MVC	3 min	18	Diabetic neuropathy	Heat	HPI TSPh	Hand Forearm	—	↓HPI and TSPh (if no pain) No changes (if pain)	2014	Knauf and Koltyn^90^
Isometric	Handgrip	25% MVC	3 min	64	Menstrual pain	Pressure	PPT		Forearm Shin	↑PPTs	2018	Travers et al.^186^
Isometric	Handgrip	30% MVC	90 seconds	12	Fibromyalgia	Pressure Heat	PPT HPI	Forearm	Forearm	↓PPTs ↑HPI (hyperalgesia)	2005	Staud et al.^177^
Isometric	Knee extension	20%–25% MVC	Fatigue	14	Fibromyalgia	Pressure	PPT	Thigh	—	↓PPT (hyperalgesia)	1996	Kosek et al.^100^
Isometric	Knee extension	10%–15% MVC	Fatigue	17	Fibromyalgia	Pressure	PPT	Thigh	Shoulder	↑PPT (shoulder)	2007	Kadetoff and Kosek^82^
Isometric	Knee extension	50% MVC	Fatigue	66	Knee OA	Pressure	PPT	Thigh	Shoulder	↑PPTs	2013	Kosek et al.^102^
Isometric	Knee extension	50% MVC	Fatigue	47	Hip OA	Pressure	PPT	Thigh	Shoulder	↑PPTs	2013	Kosek et al.^102^
Isometric	Knee extension	30% MVC	90 seconds	61	Chronic MSK pain	Pressure	PPT PTTol TSPp	Thigh	Arm Shoulder Lower leg	↑PPTs ↑PPTol	2016	Vaegter et al.^197^
Isometric	Knee extension	30% MVC	90 seconds	14	Knee OA	Pressure	PPT PTTol	Thigh	Arm Shoulder Lower leg	↑PPTs	2017	Vaegter et al.^194^
Isometric	Knee extension	10% MVC	5 min	40	Knee OA	Pressure	PPT	Thigh Knee	Forearm	↑PPTs (if normal CPM) ↓PPTs (if abnormal CPM)	2017	Fingleton et al.^40^
Isometric Isometric	Knee extension Knee extension	30% MVC 30% MVC	Fatigue 5 min	130 46	Fibromyalgia RA	Pressure Pressure	PPT PPT	— Thigh	Shoulder Shoulder	↑PPT ↑PPTs	2017 2018	Tour et al.^185^ Lofgren et al.^117^
Isometric	Knee extension	70% MVC	5 × 45 seconds	21	Patellar tendinopathy	Pressure clinical	Pain intensity during SLS PPT	Knee Shin	Forearm	↓Pain intensity ↑PPT shin	2019	Holden et al.^70^
Isometric	1. Knee extension 2. Shoulder rotation	20%–25% MVC	Fatigue	20	Shoulder pain	Pressure	PPT	Thigh Shoulder	Shoulder Thigh	↑PPTs (during knee extension)	2010	Lannersten and Kosek^107^
Isometric	1. Knee extension 2. Shoulder rotation	20%–25% MVC	Fatigue	20	Fibromyalgia	Pressure	PPT	Thigh Shoulder	Shoulder Thigh	No hypoalgesia	2010	Lannersten and Kosek^107^
Isometric	Shoulder abduction	1 kg	Fatigue	19	Chronic shoulder pain	Pressure	PPT	Shoulder	—	↑PPT	2003	Persson et al.^148^
Isometric	Shoulder abduction	Weight of arms	Fatigue	22	Fibromyalgia	Pressure	PPT	Shoulder	Shin	↓PPT shin (hyperalgesia)	2012	Ge et al.^48^
Isometric	Shoulder abduction	20%–25% MVC	5 min	24	Shoulder pain	Pressure	PPT	Shoulder	Thigh Shin	↑PPTs	2016	Kuppens et al.^105^
Isometric	Squat	70% MVC	1 exercise 5 × 45 sec repetitions	6	Patella tendinopathy	Clinical	Pain intensity during SLS	—	—	↓Pain intensity	2015	Rio et al.^158^
Isometric	Tooth clenching	—	Fatigue	20	TMD	Pressure	PPT	Jaw	Forearm	↑PPT jaw	2019	Lanefelt et al.^106^
Isometric	Wall squat	Bodyweight	3 min	21	WAD	Pressure	PPT	—	Neck Shin	↑PPTs	2017	Smith et al.^172^

The table is organized according to exercise type, exercise form, pain test modality, and year of publication.

DOMS, delayed-onset muscle soreness; HPI, heat pain intensity; HPT, heat pain threshold; HRmax, maximum heart rate; MVC, maximal voluntary contraction; PPI, pressure pain intensity; PPT, pressure pain threshold; PPTol, pressure pain tolerance; RM, repetition maximum; RPE, rating of perceived exertion; SLS, single-leg stand; TSPh, temporal summation of heat pain; TSPp, temporal summation of pressure pain; VO_2_max, maximal aerobic capacity.

### 3.1. Factors related to lack of exercise-induced hypoalgesia

Individuals with facilitated central pain mechanisms, which are commonly observed in several chronic musculoskeletal pain conditions,^121^ often report reduced hypoalgesia after exercise. Vaegter et al.^197^ observed reduced EIH after submaximal isometric exercise and after bicycling exercise in chronic pain patients with high widespread pain sensitivity compared with patients with low pain sensitivity. In addition, in high pain-sensitive patients, an increase in temporal summation of pain was observed after aerobic exercise^177,197^ possibly mimicking the pain flare-up after exercise reported in clinical practice by some individuals with widespread chronic pain.^24^ Also, Fingleton et al.^40^ observed reduced pressure pain thresholds (hyperalgesia) after both aerobic and isometric exercises in individuals with knee osteoarthritis who demonstrated an impaired CPM response. By contrast, pain thresholds increased in knee osteoarthritis individuals with a normal CPM response suggesting that patients with impaired CPM, which is also a common finding in individuals with chronic pain,^114,121^ may have less acute hypoalgesic effect from exercise.

**Figure 1.**
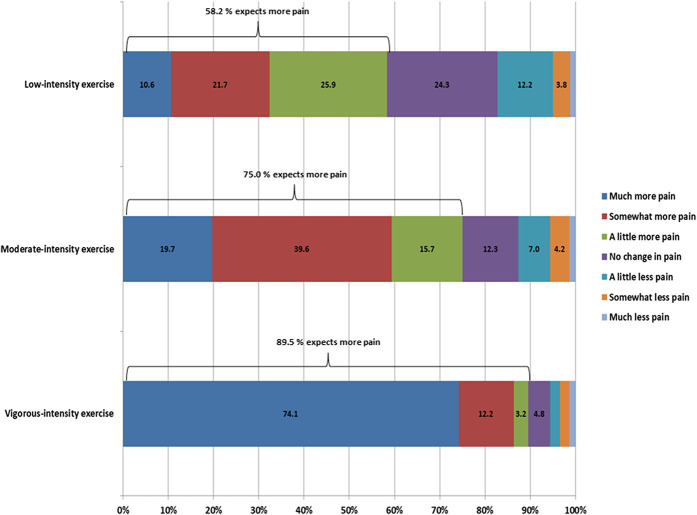
Expectations about the effects of low-intensity exercise, moderate-intensity exercise, and vigorous-intensity exercise on pain reported by patients (n = 500) referred for interdisciplinary pain treatment at a University Hospital Pain Center in Denmark (unpublished data from the clinical pain registry, PainData).

Another possible explanation for the lack of hypoalgesia after exercise often observed in individuals with chronic pain is that the exercise dose–response relationship is different in individuals with chronic pain compared with pain-free subjects. Newcomb et al.^136^ observed a larger EIH response in individuals with fibromyalgia after 20 minutes of aerobic exercise at a preferred intensity (45% of maximal heart rate) compared with a prescribed and higher-intensity aerobic exercise (60%–75% of maximal heart rate). Similarly, Coombes et al.^20^ showed that isometric exercise above but not below an individual's pain threshold increased pain responses to exercise in people with lateral epicondylalgia. These results could indicate that lower-intensity exercise creates less input to facilitated central pain mechanisms resulting in a net balance of pain inhibition and a reduction in the pain sensitivity after exercise. This may be different for chronic exercise, however, where a small benefit of painful over nonpainful exercise has been observed, albeit for clinical pain at baseline as opposed to experimental pain in the immediate post-exercise period.^173^ Other possible explanations for reduced EIH include use of opioids and negative expectations about the effect of exercise. Interactions between EIH mechanisms and the use of analgesics may affect the response to exercise. Individuals treated with opioids report less CPM,^155^ and reduced effects of opioids have been reported in animals after long-term exercise.^174^ As observed in pain-free individuals, negative expectations are associated with the hypoalgesic response after exercise. Interestingly, most patients with chronic pain referred to multidisciplinary pain treatment do not expect exercises to cause less pain; on the contrary, the majority expects more pain after exercise (Fig. 1).

### 3.2. Regular exercise and pain

Regular exercise is guideline recommended treatment for a wide range of chronic pain conditions.^49,146^ Regular exercise is safe and generally well accepted by individuals with mild to moderate chronic pain; however, the effects on pain and pain sensitivity are somewhat conflicting, and the level of evidence for a positive effect is generally low.^49^ Clinically relevant reductions in pain and pain sensitivity are often observed after 8 to 12 weeks of exercise therapy in individuals with knee or hip osteoarthritis,^170^ but randomized controlled trials often observe smaller effects with pain reductions of less than 10 on a 100-point numerical rating scale^62^ or even no change in pain after exercise therapy compared with passive sham therapy.^8^

To the best of our knowledge, only 2 studies have investigated whether habitual physical activity levels predict pain responses to acute exercise in individuals with chronic pain. Coriolano et al.^21^ found that people with knee osteoarthritis who self-reported more physical activity experienced less exacerbation in pain after completing performance-based tests and a physiological test (submaximal arm ergometer test). In people with fibromyalgia, Umeda et al.^187^ showed that participants who were more physically active reported a smaller increase in ratings of muscle pain intensity during isometric handgrip exercise. Taken together, these results suggest that being more physically active is associated with reduced pain responses to acute exercise in individuals with chronic pain. These results are consistent with cross-sectional data showing negative associations between fitness and pain (ie, more fitness, less pain) in people with fibromyalgia^77^ and knee osteoarthritis (Jones et al., in review) as well as longitudinal data showing benefit of longer periods of regular exercise training on reducing pain in individuals with chronic pain.^49^

## 4. Underlying mechanisms of exercise-induced hypoalgesia in humans

There are numerous biological and cognitive factors that contribute to pain, so changes in any one or more of these by acute exercise could account for EIH. It is not clear, however, what these mechanisms are or whether the mechanisms are similar or distinct between healthy individuals and individuals with chronic pain. The contrasting magnitude of EIH between pain-free individuals and individuals with chronic pain^130^ suggests that the mechanisms of EIH are disrupted in individuals with chronic pain. That is, some aspect of chronic pain (eg, inflammation, sensitization, and fear of movement) interferes with the normal hypoalgesic effect of acute exercise. These potential mechanisms will be described in more detail hereafter.

### 4.1. Opioid and cannabinoid systems

The most commonly proposed mechanism of EIH is enhanced descending inhibition by activation of the opioid and cannabinoid systems. The contraction of skeletal muscle increases the discharge of mechanosensitive afferents (ie, A-delta and C-fibres) which, in turn, activates central descending opioid pain pathways.^29,184^ Exercise also increases the release of endogenous cannabinoids. These opioid and cannabinoid pathways have receptors throughout the peripheral and central nervous systems that can produce analgesia when stimulated.^29,184^

Human studies investigating the role of opioids and cannabinoids in EIH have yielded equivocal findings. For example, opioid antagonists such as naloxone and naltrexone have been shown to increase, decrease, or have no effect on EIH.^30,31,74,94,140^ Moreover, correlations between EIH and exercise-induced changes in plasma concentrations of beta-endorphins and endocannabinoids are not always observed.^94,139,165^ A limitation of these human investigations is that they are more constrained than rodent studies in their ability to investigate whether opioids and cannabinoids are acting through peripheral and/or central actions to influence pain after exercise; however, there is some evidence that blocking blood flow to a limb during exercise attenuates EIH in pain-free individuals, suggesting that peripheral factors are important.^79^

### 4.2. Stress-induced hypoalgesia

Exercise-induced hypoalgesia might also be a form of stress-induced analgesia, related to the release of various stress hormones during exercise. However, evidence to support this in humans is mixed. For example, EIH is related to increases in growth hormone during exercise,^149^ but another study found that the suppression of exercise-induced growth hormone release by cyproheptadine had no effect on EIH.^86^ Dexamethasone, a steroid medication, has been shown to attenuate EIH by reducing secretion of adrenocorticotropin^88^; however, other studies have found no effect of dexamethasone on pain in healthy individuals.^209^ A small pilot study of 7 healthy individuals showed that exercise-induced changes in neuropeptide Y, allopregnanolone, pregnenolone, and dehydroepiandrosterone were related to EIH.^167^ However, because concentrations of these substances were only measured in the plasma, it is not clear whether they were acting through peripheral or central mechanisms to influence pain. Moreover, because this was only a small pilot study, more studies are needed to confirm the findings.

### 4.3. Cardiovascular systems

Exercise-induced changes in the cardiovascular system have also been proposed as a mechanism of EIH. That is, elevations in blood pressure by exercise are thought to attenuate pain through baroreceptor-related mechanisms (ie, the activation of arterial baroreceptors by exercise subsequently activates pain-related brain areas involved in pain modulation). Although it is true that people with high blood pressure are less sensitive to pain (ie, hypertension-associated hypoalgesia),^161^ there is currently little evidence that acute changes in blood pressure by exercise are related to EIH.^28,157,189,190^ Moreover, acute increases in blood pressure by exercise could not account for the persistence of EIH after exercise (eg, 15 minutes after exercise cessation^210^ because blood pressure would have presumably returned to baseline, or indeed be lower, by this time).

### 4.4. Central pain modulatory systems

The influence of exercise on reducing the sensitivity of the central nervous system has also been explored as a mechanism of EIH. These studies show that acute exercise can reduce temporal summation^96,131,196,206^ and increase thresholds to elicit the nociceptive withdrawal reflex,^55^ although there is some evidence contrary to the latter observation.^125^ These results imply that exercise can reduce pain through reductions in central nervous system sensitivity at spinal and supraspinal levels, but exactly where in the nociceptive pathway these changes occur is not known. Improved efficacy of descending inhibitory pathways by exercise has been studied as a mechanism of EIH as well, but there is little direct evidence to support this. For example, Alsouhibani et al.^2^ observed a decrease in the CPM response after exercise, Meeus et al. found no effect of aerobic exercise on CPM in healthy individuals,^122^ and Ellingson et al.^36^ showed that EIH was comparable for nonpainful and painful exercise, although the latter should have evoked a larger “pain inhibits pain” effect. A few studies have found small positive correlations between conditioned pain modulation and EIH^13,112,198^ suggesting that the 2 may share similar mechanisms; however, EIH is usually somewhat smaller in magnitude but more enduring than conditioned pain modulation so the 2 are likely distinct.^112,195^

### 4.5. Psychological contributing factors

Changes in pain cognition might also account for some of the effect of acute exercise on pain. It has been shown that exercise can reduce ratings of pain unpleasantness in the absence of a change in ratings of pain intensity,^80^ suggesting that alterations in the appraisal of noxious stimuli contribute to EIH. Cognitive and psychosocial factors including pain self-efficacy, coping strategies, fear of pain, and stress are known to underlie some of the difference in pain between athletes and nonathletes,^53,75,142^ but their relation to EIH is less clear. For example, several studies have shown that individuals with higher levels of catastrophizing experience less EIH,^16,131,207^ although this is not always observed and correlations between EIH and other psychosocial factors (eg, fear of pain, pain attitudes, and anxiety) seem negligible.^112,201^ Therefore, the contribution of cognitive factors to EIH remains poorly understood but seems limited. More studies are needed to investigate whether these cognitive factors are related to EIH and, more importantly, whether they can be manipulated to augment it.^81^

### 4.6. Impaired EIH: disrupted or distinct mechanisms

The mechanisms of EIH in individuals with chronic pain are equally if not more unclear. Because exercise has such varying effects on pain within and between individuals with chronic pain, it is difficult to determine whether there is a consistent mechanism that contributes to changes in pain with acute exercise. Moreover, it is not clear if the mechanisms of EIH in individuals with chronic pain are the same as pain-free individuals and are just disrupted, or whether separate mechanisms related to the presence of chronic pain are involved as well.

The fact that EIH can occur at exercised and remote sites in individuals with chronic pain shows that EIH is not always disrupted in these individuals.^38,136,197^ However, there are also several demonstrations that exercise with a painful joint or muscle can either diminish EIH compared to when a nonpainful body part is exercised (ie, exercise of the upper limb in people with knee osteoarthritis, but pain measurement in the lower limb)^17^ or, worse, can increase pain.^19,20,107,177^ These results are both opposite to what is normally seen in pain-free individuals where EIH is usually greatest for the exercised body part. Therefore, the results of the above studies provide some evidence that compared to healthy individuals, the mechanisms of EIH in individuals with chronic pain are both similar and distinct. However, because the mechanisms of EIH are still poorly understood in both groups, there is little direct evidence to support this.

Regarding mechanisms of EIH that may be similar, but disrupted, in individuals with chronic pain compared to pain-free individuals, altered excitability of the central nervous system after exercise is perhaps the most obvious. In pain-free individuals, acute exercise reliably reduces temporal summation,^96,196,206^ whereas the opposite effect has been observed in individuals with chronic pain.^197,206^ By contrast, one of the few studies to combine acute exercise with analgesic medication showed that paracetamol and placebo had comparable effects on temporal summation and conditioned pain modulation after exercise in pain-free individuals and individuals with chronic pain.^122^ Because paracetamol is a predominantly central acting agent that can affect opioids, cannabinoid and serotonergic pathways,^168^ this finding provides little support to the notion that exercise reduces pain through central changes in these pathways or that differences in the sensitivity of these pathways through exercise accounts for the greater EIH in pain-free individuals compared to individuals with chronic pain. More studies using drugs with less ubiquitous effects would be useful to further investigate how different substances are involved in EIH in humans and whether these differ between pain-free individuals and individuals with chronic pain.

As for mechanisms of EIH that might be distinct between pain-free individuals and individuals with chronic pain, reductions in inflammation by acute exercise are one such possibility. Inflammation plays a key role in the pathogenesis of several chronic pain states, so it is possible that reductions in inflammation by exercise may reduce pain in these individuals. However, the results of studies examining the effect of acute exercise on inflammation in individuals with chronic pain are mixed and the relation between the changes in inflammatory markers and pain has seldom been explored. Moreover, differences in the exercise-induced changes in inflammatory markers between individuals with chronic pain and pain-free individuals were only sometimes, but not always, observed. Therefore, it remains unclear to what extent EIH is related to acute changes in inflammation by exercise in individuals with chronic pain or whether this is a distinct mechanism of EIH in these populations. Another possibility is opioid-induced hyperalgesia. As already mentioned, interactions between EIH mechanisms and the use of analgesics may affect the response to exercise. Individuals treated with opioids report less CPM,^155^ and reduced effects of opioids have been reported in animals after long-term exercise.^174^ This may be explained by opioid-induced hyperalgesia which, paradoxically, leads to a reduction in central opioid receptor availability^60^ and hence less potential to modulate pain through opioidergic mechanisms (as shown in pain-free individuals.^152^

Psychosocial and cognitive factors are heavily implicated in the development and persistence of chronic pain.^34^ These same cognitive factors influence responses to experimental noxious stimuli in pain-free individuals as well,^150^ but their relation to EIH has seldom been examined, particularly in individuals with chronic pain. Accordingly, it is still not known whether cognitive factors are directly involved in EIH, or, perhaps more importantly, whether they can be manipulated to influence pain responses to exercise. Although there is some evidence to support this in pain-free individuals,^81^ it remains to be determined whether preceding exercise with education can also influence EIH in individuals with chronic pain in whom negative expectations about pain and exercise are more prevalent and therefore likely harder to change. It may be that, because of their more entrenched negative beliefs about pain and exercise, more intensive education is required in individuals with chronic pain to produce the same effect. Some combination of pain neuroscience education and EIH education might also be required. Nonetheless, if the effect can be replicated in individuals with chronic pain, it could have important applications for exercise prescription in clinical practice.

Regarding regular exercise, despite the large number of studies that have shown exercise training to reduce pain in people with chronic pain,^49^ the mechanisms by which it does this is poorly understood. This is largely because many of the studies did not analyze which changes occurring with exercise (biological and/or psychological changes) were associated with the observed improvements in pain. Moreover, few of the studies investigated where in the nociceptive pathways (ie, peripheral, spinal, and/or supraspinal pathways) changes might be occurring due to exercise, which could account for the observed reductions in pain. As a result, the precise mechanisms of pain attenuation by exercise training are not known, but several possibilities exist that are likely common to individuals with chronic pain.

Improved structure and function of the musculoskeletal system is one such possibility. In people with knee osteoarthritis, chronic exercise can improve several musculoskeletal factors important in the development and progression of the disease including body mass, joint alignment, proprioception, cartilage structure and function, inflammation, and muscle strength.^6,160^ Of these possible mediators, improvements in muscle strength are the strongest contributor to the positive effect of physical exercise on improved osteoarthritis symptoms.^160^

Desensitization of the nervous system is another possibility. In humans, exercise-induced changes in biomarkers associated with nociceptive pathways have been reported (eg, inflammatory factors and neurotransmitters),^83^ but again it is not clear whether these changes reduce pain due to the peripheral or central actions of these factors. Preliminary evidence shows that exercise can normalise aberrant brain activity in people with fibromyalgia.^41^ This finding is in agreement with the results of a few cross-sectional studies showing that people with fibromyalgia who are more physically active have more typical brain responses to pain compared to less active individuals.^37,120^ However, not all studies have shown chronic exercise to attenuate aberrant brain responses in individuals with chronic pain,^126^ so the role of changes in brain activity as a mechanism of pain relief by regular exercise remains unclear.

Finally, exercise-induced improvements in mood could be another shared mediator of the positive effect of exercise on pain in individuals with chronic pain. The role of both general (eg, depression and anxiety) and pain-specific (eg, catastrophizing and self-efficacy) psychosocial processes in the development and maintenance of chronic pain is clear.^34^ Many of these psychosocial factors are positively influenced by exercise,^85,183^ so it is plausible that this could result in improvements in pain either directly or indirectly through changes in both the sensory and emotional aspects of pain.

## 5. Implications and future perspectives

### 5.1. Clinical implications

Most types of exercise can reduce pain sensitivity at exercising and nonexercising muscles in pain-free individuals, with a larger hypoalgesic response at the exercising muscles. In individuals with chronic pain, the hypoalgesic response after exercise is less consistent; however, in addition to other well-documented physical and mental health benefits related to exercise, exercise can sometimes induce hypoalgesia in individuals with chronic pain. Regarding exercise prescription in clinical settings, it may be worth considering: (1) that exercising nonpainful body areas if possible as well as using low-intensity exercises such as walking may be useful as a first step, (2) that individuals' beliefs, expectations, and exercise preference should be assessed before exercise prescription to minimize the risk of a poor outcome, and (3) that these beliefs and expectations could be modified through education or other interventions to improve pain responses to exercise in people with chronic pain. There is some evidence that combining exercise training and education has superior effects compared to exercise alone in individuals with chronic pain,^15,153^ but this is yet to be properly explored in the context of pain responses to a single bout of acute exercise in individuals with chronic pain.

### 5.2. Implications for future exercise-induced hypoalgesia studies

In addition to the above-mentioned implications, we also propose several methodological recommendations for future studies of EIH. First, studies should use a randomized controlled design (parallel or crossover), or at the very least include a control group/condition. This is because the causal effects of exercise on pain are best inferred from randomized controlled trials. As shown in Tables 1 and 2, there have been well over 150 studies of EIH in pain-free individuals and individuals with chronic pain. However, the minority of these used a randomized controlled design or a nonrandomized controlled design. Instead, EIH was often investigated using a single-arm pre-post design. A major limitation of this type of study design is that the effects of habituation to noxious stimuli, as well as statistical phenomena such as regression to the mean, are not accounted for. To truly determine whether a single bout of exercise causes a reduction in pain, randomized controlled trials are needed. Second, it is important that these randomized controlled trials use large(r) sample sizes. The majority of EIH studies are small (n ≤ 50), and it is well documented that small studies are inherently biased to find larger effects.^26^ Hence, most studies of EIH probably overestimate the effect of exercise on pain. Consequently, despite the enormous amount of EIH studies to date, the true effect of a single bout of exercise on pain is still unknown. Larger randomized controlled trials, of which there are currently very few, are clearly needed to determine this.

As evident in Tables 1 and 2, there is substantial heterogeneity in methodology used in EIH studies, making it difficult to synthesise the results of this vast literature. Therefore, we also recommend that future EIH studies share a somewhat common methodology so that the results between studies can be more easily compared. To this end, it may be useful for future studies to share a common method of pain assessment. Pressure pain thresholds at local and remote sites may be the most appropriate because these have been studied most often and do not require expensive equipment (although they may be more prone to experimenter bias if using handheld algometry). It would also be of benefit to include assessment of both experimental and clinical pain in individuals with chronic pain to better understand the effects of exercise on “real life” pain. Moreover, it may be useful to prescribe and report exercise using a common index so that the amount of work performed can be quantified. This would help clarify the dose–response effect of exercise on pain, a result that may have important clinical implications such as determining the minimal effective dose with respect to hypoalgesia for each mode of exercise as well as identifying volumes and/or intensities of exercise that may be more likely to exacerbate pain in individuals with chronic pain. Finally, Lee et al.^109^ recently outlined several issues in clinical pain research including transparency, underpowered studies, and researcher degrees of freedom. The use of preregistration and registered reports, data sharing, and greater adherence to reporting guidelines were suggested as areas for improvement and we believe that EIH studies would benefit from adopting these recommendations.

## Disclosures

The authors have no conflicts of interest to declare.
